# ^131^I-LNTH-1095 Radioligand Therapy plus Enzalutamide versus Enzalutamide Alone in Men with PSMA-Avid Metastatic Castration-Resistant Prostate Cancer: A Phase II Study

**DOI:** 10.1158/1078-0432.CCR-25-4948

**Published:** 2026-03-04

**Authors:** Evan Y. Yu, Vivek Narayan, Giuseppe Esposito, Russell Szmulewitz, Yang Lu, Michael B. Lilly, Jeremie Calais, Gennady Bratslavsky, Yusuf Menda, Minal Vasanawala, Frédéric Pouliot, David Laidley, Neil Fleshner, Fred Saad, Jean-Claude Provost, Iryna Teslenko, Nand Kishore Rawat, Gary Ulaner

**Affiliations:** 1 Fred Hutchinson Cancer Center, University of Washington, Seattle, Washington.; 2 University of Pennsylvania, Philadelphia, Pennsylvania.; 3 Medstar Georgetown University Hospital, Washington, District of Columbia.; 4 Duchossois Center for Advanced Medicine, University of Chicago, Chicago, Illinois.; 5 The University of Texas MD Anderson Cancer Center, Houston, Texas.; 6 Medical University of South Carolina, Charleston, South Carolina.; 7 University of California, Los Angeles, Los Angeles, California.; 8 SUNY Upstate Medical University, Syracuse, New York.; 9 University of Iowa, Iowa City, Iowa.; 10 VA Palo Alto Health Care System, Palo Alto, California.; 11 Centre Hospitalier Universitaire de Québec, Université Laval, Québec, Canada.; 12 London Health Sciences Centre, London, Canada.; 13 Division of Urology, University of Toronto, Toronto, Canada.; 14 Centre Hospitalier de l’Université de Montréal, Montréal, Canada.; 15 Lantheus, Bedford, Massachusetts.; 16 Hoag Family Cancer Institute, Irvine, California.; 17 University of Southern California, Los Angeles, California.

## Abstract

**Purpose:**

The phase II ARROW study was designed to evaluate radioligand therapy (RLT) with ^131^I-LNTH-1095, an iodine-131–labeled small molecule targeting prostate-specific membrane antigen (PSMA), in combination with enzalutamide in subjects with metastatic castration-resistant prostate cancer after progression on prior abiraterone therapy.

**Patients and Methods:**

Men ≥18 years with PSMA-positive prostate cancer (PSMA PET tracer uptake >1× liver SUV_mean_ in all CT-measurable lesions) were randomly assigned 2:1 to ^131^I-LNTH-1095 (4 cycles of 3.7 GBq/dose every 8 weeks) + enzalutamide (160 mg orally once daily) versus enzalutamide alone. The primary endpoint was PSA_50_ response. Secondary endpoints included radiographic progression-free survival (rPFS), objective response rate, overall survival (OS), and safety.

**Results:**

Of 177 screened subjects, 120 were randomly assigned (80: ^131^I-LNTH-1095 + enzalutamide; 40: enzalutamide monotherapy). PSA_50_ response was 62.9% [95% confidence interval (CI), 50.5–74.1] for ^131^I-LNTH-1095 + enzalutamide versus 31.3% (16.1–50) for enzalutamide alone (*P* = 0.003). The median rPFS was 14.0 months (95% CI, 8.64–18.20) for ^131^I-LNTH-1095 + enzalutamide versus 11.5 months (2.79–18.43) for enzalutamide alone (*P* = 0.10). The incidence of grade ≥3 treatment-emergent adverse events (TEAE) was 65.8% for ^131^I-LNTH-1095 + enzalutamide versus 41% for enzalutamide monotherapy; the most frequent TEAEs were fatigue (75% vs. 53.8%), nausea (59.2% vs. 33.3%), thrombocytopenia (51.3% vs. 0%), and decreased appetite (48.7% vs. 17.9%), respectively. Two deaths in the ^131^I-LNTH-1095 + enzalutamide group were considered treatment-related. The study was not powered to detect rPFS and OS differences.

**Conclusions:**

^131^I-LNTH-1095 + enzalutamide was associated with a statistically significant improvement in PSA_50_ response compared with enzalutamide alone despite a lower dosing schedule (4 cycles of 3.7 GBq/dose every 8 weeks) than the other approved PSMA RLT agents. Grade ≥3 adverse events were more frequent with combination therapy, particularly hematologic toxicity.

**Translational Relevance**

Prostate cancer is the most common cancer among men. Despite significant advances in treatment options, metastatic disease remains incurable. ^131^I-LNTH-1095 is a candidate, non–FDA approved prostate-specific membrane antigen (PSMA)–targeted radioligand therapy (RLT) under development as a treatment option for subjects with metastatic castrate-resistant prostate cancer (mCRPC). This study investigated the efficacy and safety of this agent when using both a unique radioisotope payload (iodine-131) and limited treatment regimen (a maximum of four cycles) in combination with enzalutamide. This combination increased the likelihood of PSA_50_ response among mCRPC subjects compared with subjects receiving enzalutamide alone. In addition, ^131^I-LNTH-1095 was well tolerated, with a safety profile consistent with known RLT effects. Future studies are warranted to reevaluate the optimal radioisotope for potential therapeutic use with this unique PSMA-targeted ligand.

## Introduction

Prostate-specific membrane antigen (PSMA), a type II membrane glycoprotein encoded by folate hydrolase 1 (*FOLH1*), is overexpressed in most prostate cancers (1, 2). Its expression in normal tissues is mainly limited to the kidney, gastrointestinal tract, and salivary glands and minimal in other normal organs (2).Therefore, the use of radioligands targeting PSMA is attractive for imaging (favorable lesion to background ratio) and treatment (favorable therapeutic index) of prostate cancer. Multiple ligands are in development, and one therapy received FDA approval, lutetium Lu 177 vipivotide tetraxetan (^177^Lu-PSMA-617), for PSMA-positive metastatic castration-resistant prostate cancer (mCRPC; refs. 3, 4).

^131^I-LNTH-1095 (previously known as ^131^I-MIP-1095) was developed as a PSMA-targeted imaging agent and subsequently observed to selectively produce targeted cell death in prostate cancer cells (5–8). Its mechanism of action involves PSMA binding, internalization, and the subsequent emitting of β particles. The rationale for choosing this isotope was its suitable half-life and well-established therapeutic utility in other oncological areas of nuclear medicine. Enzalutamide is an androgen receptor pathway inhibitor (ARPI) used to treat prostate cancer and has been observed to enhance PSMA expression of prostate cancer cells under some conditions (9, 10). In nonclinical studies, the combination of PSMA-directed treatment modalities plus antiandrogens had synergistic cytotoxic effects against prostate cancer cells (11–13). It was these observed combined effects that served as the rationale for this study and its PSA_50_ response primary efficacy endpoint.

ARROW (NCT03939689) is a phase II, open-label, randomized clinical study that compared therapy with ^131^I-LNTH-1095 + enzalutamide versus enzalutamide monotherapy in men with PSMA-avid mCRPC that had progressed during prior treatment with the CYP17 inhibitor abiraterone.

## Patients and Methods

### Trial design and subjects

Subjects were enrolled at 23 sites in the United States and Canada. Eligible subjects were men (≥18 years) who were to receive enzalutamide after their mCRPC progressed on abiraterone and were ineligible for, or declined, taxane-based chemotherapy based on personal preference or physician opinion. The clinical benefit of enzalutamide following abiraterone in mCRPC has been demonstrated in a randomized phase II trial (14), supporting its use in this patient population. Initial diagnosis required confirmed prostate adenocarcinoma without neuroendocrine differentiation or small cell features. Metastatic disease was confirmed by bone lesions on a whole-body bone scan or soft-tissue lesions measurable per RECIST 1.1 on CT or MRI. At screening, subjects with castrate status (serum testosterone ≤50 ng/dL) required a life expectancy of ≥6 months and Eastern Cooperative Oncology Group (ECOG) performance status 0 to 2, and had to provide signed informed consent and agree to comply with protocol requirements.

Key exclusion criteria included antitumor therapy within 4 weeks of randomization; prior chemotherapy (except for taxane-based chemotherapy for castration-sensitive prostate cancer); radiopharmaceuticals (e.g., strontium-89 and radium-223) within 6 months prior to randomization; prior PSMA-targeted radioligand therapy (RLT); enzalutamide use for >7 days before consent; active non–prostate cancer malignancy; or investigator-determined conditions compromising study participation.

This study was approved by the Institutional Review Board/Research Ethics Board at each participating institution and conducted in accordance with the Declaration of Helsinki and the International Council on Harmonization Guidelines for Good Clinical Practice.

### Study monitoring/oversight

An independent data monitoring committee assessed the safety and efficacy of the trial’s interventions and monitored the trial’s overall conduct.

### PSMA PET screening with ^18^F-piflufolastat

Subjects underwent PET with ^18^F-piflufolastat [333 MBq (9 mCi)] at screening, at the end of cycle 1, and at the end of treatment (EOT). Randomization required significant PSMA uptake as determined by central review in all prostate cancer lesions (SUV_max_ >1× liver SUV_mean_), except for PSMA-negative soft-tissue lesions (<1 cm, short axis), lymph node lesions (<1.5 cm, short axis), bone lesions with a soft-tissue component (<1 cm, short axis), or without a soft-tissue component (any size).

All ^18^F-piflufolastat PET/CT scanners used in this study were qualified prior to dosing in accordance with the procedures outline in the imaging manual. All scans were submitted in Digital Imaging and Communication in Medicine (DICOM) format to the Core Imaging Laboratory, where they were reviewed for image quality and adherence to specifications. DICOM header data were additionally assessed to ensure SUV accuracy.

### Stratification by risk group

Prior to randomization (2:1 ^131^I-LNTH-1095 + enzalutamide or enzalutamide monotherapy), eligible subjects were stratified by protocol-defined risk group (15) based on laboratory results at screening [intermediate risk: hemoglobin (Hgb) ≥11 g/dL, lactate dehydrogenase (LDH) <262 IU/L, and alkaline phosphatase (ALP) <414 IU/L; high risk: Hgb <11 g/dL, LDH ≥262 IU/L, or ALP ≥414 IU/L].

### Study treatment

All subjects were prescribed enzalutamide (160 mg orally once daily). The RLT group also received up to four cycles of ^131^I-LNTH-1095 (≤3.7 GBq/dose every 8 weeks up to four doses). The selection of a 3.7 GBq (100 mCi) activity was based on prior dosimetry data (7) showing that absorbed doses to critical organs remained within acceptable limits (16). Furthermore, ^131^I-LNTH-1095 therapy with doses up to 200 mCi was well tolerated, with no dose-limiting toxicities (DLT) observed in patients with mCRPC (17). More recently, Laccetti and colleagues (18) evaluated ^131^I-LNTH-1095 at lower doses (50–75 mCi) and reported favorable biodistribution and dosimetry, supporting its utility in mCRPC (18).

Both agents were initiated on the same day to avoid delaying RLT and to reflect a treatment strategy consistent with real-world clinical practice. For thyroid blockage, potassium iodide was administered 24 hours before ^131^I-LNTH-1095 and continued for 10 days. Using a two-tiered dosing approach, before cycle 3, subjects received a 370 MBq dose of ^131^I-LNTH-1095 for dosimetric imaging, which was assessed by central review. This timing was selected to evaluate cumulative organ exposure after initial treatment and guide dosing for subsequent cycles. The maximum cumulative dose/patient was determined per International Commission on Radiological Protection (ICRP) limits: kidneys (18 Gy), liver (30 Gy), lungs (18 Gy), and bone marrow (10 Gy; ref. 15). Subsequent doses (2.775 GBq or 3.7 GBq) and number of cycles were determined by dosimetry, subject to recovery from any DLTs. This dosimetry-based treatment facilitated individualized treatment while preserving patient safety. After EOT (week 53), follow-up visits were scheduled every 13 weeks until study completion.

### Study assessments and endpoints

Visits for efficacy and safety assessments were conducted, including prostate-specific antigen (PSA) measurements at baseline and weeks 5, 9, 13, 17, 21, 25, 29, 36, 42, 47, and 53. Radiographic assessment was performed on day 1 of each cycle or whenever progression was suspected. Following treatment completion or early discontinuation, survival follow-up included phone-based collection of survival data, adverse events (AE) of special interest, and new anticancer therapy data for at least 1 year or until study completion.

The primary efficacy endpoint was PSA_50_ response, defined as the first occurrence of a ≥50% decline in PSA from baseline confirmed ≥3 weeks later, and selected for its established role in evaluating antitumor activity in early-phase mCRPC trials (19).

The objective response rate (ORR) was defined as the proportion of subjects who had a partial response (PR) or complete response (CR) based on RECIST 1.1 (20) criteria for subjects with measurable soft-tissue disease at baseline. Radiographic progression-free survival (rPFS) was defined as the time from randomization to first occurrence of radiographic progression [based on RECIST 1.1 and Prostate Cancer Working Group 3 (PCWG3) criteria; ref. 21] or death from any cause. Overall survival (OS) defined as time from randomization to death or last date confirmed alive, time to PSA progression defined by PCWG3 criteria, and time to new anticancer treatment was also determined. Duration of response (DoR) was the time from first date of CR or PR to first occurrence of radiographic progression (based on PCWG3-modified RECIST 1.1) or unequivocal clinical progression.

Exploratory efficacy endpoints include change in ^18^F-piflufolastat uptake (SUV_max_) from baseline to EOT (week 53). Exploratory analyses, including a summary of ^18^F-piflufolastat SUV_max_ in PSA_50_ responders versus nonresponders and by number of ^131^I-LNTH-1095 therapy cycles at baseline and EOT, were determined.

Additional exploratory variables include time from randomization to first symptomatic skeletal event, incidence of pain progression [increase of ≥30% from baseline in the Brief Pain Inventory Short Form (BPI-SF) pain intensity score], change in Functional Assessment of Cancer Therapy—Prostate (FACT-P) Questionnaire score, change in score on the 12-item Short Form Survey (SF-12), EuroQol Group health-related quality of life assessment (EQ-5D-5L and EuroQoL VAS), change in the automated bone scan index (aBSI), and change in ECOG performance status.

Safety variables [extent of exposure to study drugs, treatment-emergent AEs (TEAE)], changes in laboratory parameters, physical examination findings, vital signs, and electrocardiograms were evaluated in the safety population (subjects who received ≥1 dose of the study drug).

### Statistical analyses

Assuming a PSA_50_ response of 30% for enzalutamide monotherapy (22) and ≥59% for ^131^I-LNTH-1095 + enzalutamide, a minimum of 102 subjects would be required to yield 80% power (two-sided α = 0.05), based on a 2:1 randomization ratio (68 to ^131^I-LNTH-1095 + enzalutamide; 34 to enzalutamide monotherapy). With a 15% dropout allowance, 120 subjects were randomly assigned (80 to ^131^I-LNTH-1095 + enzalutamide; 40 to enzalutamide monotherapy).

ORR was analyzed using a two-sided *χ*^2^ test to compare proportions between treatment groups, with a type I error of 0.05. If >20% of expected cell frequencies were <5, then a two-sided Fisher exact test was performed. Secondary endpoints were analyzed using a two-sided log-rank test, stratified by risk category (intermediate risk vs. high risk).

## Results

### Patient population/disposition

**Figure 1.**
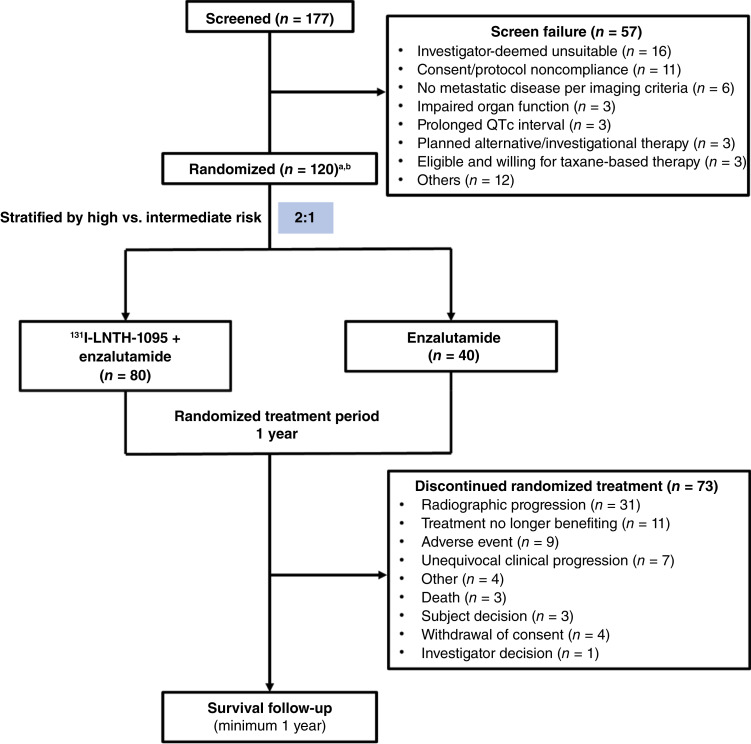
Study design and patient disposition. ^a^Four subjects met PSMA avidity but were not randomly assigned, and one subject was not PSMA imaged but was randomly assigned. ^b^Two subjects were randomly assigned and later found to be screen failures.

The study commenced in May 2019 and concluded in September 2023. Of 177 screened subjects, 57 were ineligible (Fig. 1). The most common screen failures were due to investigator-deemed unsuitability (*n* = 16) and consent/protocol noncompliance (*n* = 11). Among 132 subjects who underwent PSMA imaging with ^18^F-piflufolastat, 123 met avidity criteria and 6 did not; 3 subjects were unevaluable (Supplementary Table S1; Fig. 1). The full analysis set included 120 randomly assigned subjects (80: ^131^I-LNTH-1095 + enzalutamide; 40: enzalutamide monotherapy). The safety set comprised 115 subjects who received ≥1 dose (76: ^131^I-LNTH-1095 + enzalutamide, 39: enzalutamide).

A– total of 42 subjects (29: ^131^I-LNTH-1095 + enzalutamide; 13: enzalutamide) completed the randomized treatment period. Seventy-three subjects discontinued (47: ^131^I-LNTH-1095 + enzalutamide; 26: enzalutamide), most often due to radiographic progression (25.8%), lack of treatment benefit (9.2%), or an AE (7.5%).

Treatment arms were demographically and clinically balanced. In the safety set (*n* = 115), 82.6% were White, 9.6% Black/African American, 1.7% Asian, and 4.3% Hispanic/Latino. Baseline characteristics, including tumor burden and disease history, and representativeness of study participants are summarized in Supplementary Tables S2–S5.

Overall, the median time since the initial diagnosis of the subject’s prostate cancer was 54.5 months (range, 1–257 months). Notably, the median time from initial prostate cancer diagnosis to study treatment was longer in the ^131^I-LNTH-1095 + enzalutamide group than in the enzalutamide monotherapy group (63.8 vs. 38.3 months), whereas the means (SDs) were comparable 76.7 (62.19) versus 64.3 (57.15) between the two groups. Additionally, the proportion of subjects initially diagnosed with synchronous metastatic prostate cancer was lower in the ^131^I-LNTH-1095 + enzalutamide group (40.8%) than in the enzalutamide monotherapy group (53.9%).

The median treatment duration was 36.2 weeks for the ^131^I-LNTH-1095 + enzalutamide group versus 28.6 weeks for enzalutamide monotherapy. The combination group received a mean (SD) of 2.7 (1.01) therapeutic doses of ^131^I-LNTH-1095. Dose delays (median 1; range, 1–3 delays) occurred in 35 subjects (46.1%), due to supply-related issues (17; 22.4%), recovery from hematologic toxicity (7; 9.2%), or other reasons (11; 14.5%). Dose reduction (≥1) occurred in 40 (52.6%) subjects, with the majority (37 subjects) due to dosimetry assessments rather than AE. Of the 50 recorded dose reductions in 40 subjects, 43 resulted from dosimetry, 5 were due to toxicity, and 2 were due to administration irregularities. Reductions were implemented when cumulative absorbed doses to critical organs exceeded ICRP safety thresholds, based on individualized dosimetry.

### Primary endpoint: PSA_50_ response

**Figure 2.**
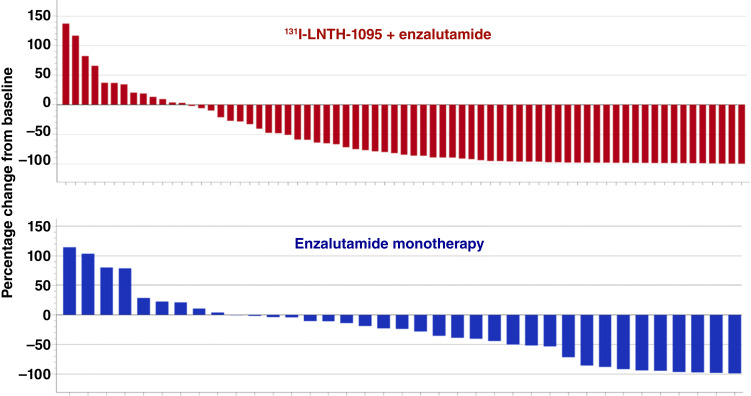
Best PSA decline from baseline (%), any measurement.

PSA_50_ response was evaluable for 102 subjects (70: ^131^I-LNTH-1095 + enzalutamide; 32: enzalutamide monotherapy), defined as a baseline PSA value and ≥2 post-dosing PSA assessments. Eighteen subjects did not meet these evaluability criteria and were excluded from the analysis. A statistically significant improvement in PSA_50_ response was observed with ^131^I-LNTH-1095 + enzalutamide: 62.9% [95% confidence interval (CI), 50.5%–74.1%] versus 31.3% (95% CI, 16.1%–50%) with enzalutamide monotherapy (Table 1). The relative risk (RR) of PSA_50_ with ^131^I-LNTH-1095 + enzalutamide versus enzalutamide monotherapy was 2 (*P* = 0.003). Figure 2 shows the waterfall plots for best decrease in PSA from baseline.

**Table 1.** Primary efficacy analysis.

Primary endpoint	^131^I-LNTH-1095 + enzalutamide (*N* = 80)	Enzalutamide monotherapy (*N* = 40)
Evaluable cases, *n*	70	32
PSA_50_,^a^*n* (%)	44 (62.86%)	10 (31.25%)
95% CI	50.5–74.1	16.1–50
OR (95% CI)^b^	3.9615 (1.5941–9.8446)	
RR (95% CI)^b^	2.0331 (1.1837–3.4920)	
*P* value	0.0025	
PSA_50_ by stratification factor	​	​
Intermediate risk^c^ (evaluable cases), *n*	55	26
PSA_50_, *n* (%)	37 (67.27%)	9 (34.62%)
High risk^d^ (evaluable cases), *n*	15	6
PSA_50_, *n* (%)	7 (46.67%)	1 (16.67%)

a PSA_50_ is the percentage of subjects who have a ≥50% reduction from baseline in serum PSA, confirmed ≥3 weeks later.

b Cochran–Mantel–Haenszel statistics for the ratio of ^131^I-LNTH-1095 + enzalutamide to enzalutamide monotherapy, adjusted for factors used at randomization.

c Hemoglobin ≥11 g/dL, LDH <262 IU/L, and ALP <414 IU/L at screening.

d Hemoglobin <11 g/dL, LDH ≥262 IU/L, or ALP ≥414 IU/L at screening.

Subgroup analyses by age, race, ethnicity, and baseline LDH demonstrated a generally consistent effect. Treatment arm differences were statistically significant (*P* < 0.05) for subjects 65 to <75 years, White, not Hispanic or Latino, and those with LDH ≤ upper limit of normal (Supplementary Table S6). A sensitivity analysis was also performed which determined the proportion of PSA responders by treatment group without adjustment for risk stratification. In that analysis, the odds ratio (OR) was 3.7231 (95% CI, 1.5274–9.0749) and the RR was 1.8510 (95% CI, 1.2608–2.7174); the difference between treatment groups was statistically significant (*P* = 0.0031).

In the ^131^I-LNTH-1095 + enzalutamide group, responders by number of ^131^I-LNTH-1095 therapy cycles were 16.7% (1/6), 44% (11/25), 87.5% (14/16), and 78.3% (18/23) for cycles 1 to 4, respectively.

### Secondary endpoints

#### ORR

Best overall response, as assessed by the local investigator in subjects with RECIST 1.1 measurable disease between baseline and the final assessment, was 16.7% (4/24; 95% CI, 6.1%–36.5%) in the ^131^I-LNTH-1095 + enzalutamide group and 17.9% (7/39; 95% CI, 8.7%–33%) in the enzalutamide monotherapy arm.

The best overall response, as per predefined protocol endpoint and assessed by the local investigator at any time between baseline and the final assessment, was 11% (7/63; (95% CI, 4.6%–21.6%) in the ^131^I-LNTH-1095 + enzalutamide group and 12% (4/33; 95% CI, 3.4%–28.2%) in the enzalutamide monotherapy arm. This difference was not statistically significant (*P* = 0.81).

#### rPFS

**Figure 3.**
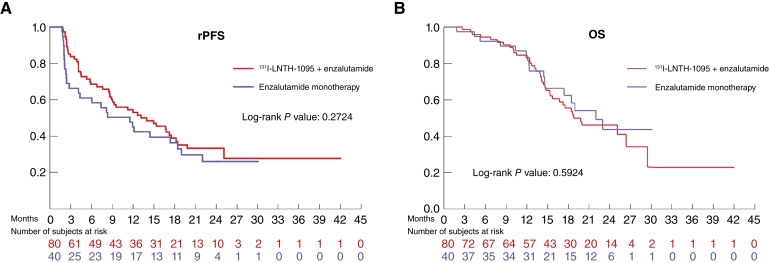
rPFS, as determined by the local investigator (**A**), and OS (**B**).

Table 2 presents rPFS results, as assessed by the local investigator. The difference between groups was not statistically significant (Fig. 3A).

**Table 2.** rPFS.

Randomized	^131^I-LNTH-1095 + enzalutamide (*N* = 80)	Enzalutamide monotherapy (*N* = 40)
rPFS	​	​
Radiographic progressive disease, *n* (%)	47 (58.8)	27 (67.5)
Censored,^a^*n* (%)	33 (41.3)	13 (32.5%)
Time to event, months, median (95% CI)	14 (8.64–18.20)	11.5 (2.79–18.43)
Event-free rate at 12 months,^b^ % (95% CI)	50 (41–64)	50 (29–60)
Log-rank *P* value^c^	0.1032	
Deaths, *n* (%)	38 (47.5)	17 (42.5)
By stratification factor	​	​
Intermediate-risk^d^ prostate cancer, *n*	63	31
Time to event, months, median (95% CI)	14.9 (8.87–25.10)	14.4 (8.15–NE)
Log-rank *P* value^e^	0.7751	
High-risk^f^ prostate cancer, *n*	17	9
Time to event, months, median (95% CI)	8.4 (2.66–16.69)	2.1 (1.74–2.79)
Log-rank *P* value^e^	0.0004	

rPFS was defined as the number of months from the date of randomization through the first occurrence of radiographic progressive disease or death, whichever came first.

a Subjects are censored at the date of the occurrence of withdrawal, study completion, or the last date that they were known to be alive.

b Event-free rates and 95% CI are estimated by the Kaplan–Meier method and Greenwood formula.

c From the log-rank test of no difference between treatment groups, stratified by prostate cancer risk group.

d Hemoglobin ≥11 g/dL, LDH <262 IU/L, and ALP <414 IU/L at screening.

e From the log-rank test of no difference between treatment groups.

f Hemoglobin <11 g/dL, or LDH ≥262 IU/L, or ALP ≥414 IU/L at screening.

#### OS

At the protocol-specified 12-month timepoint, 38 (47.5%) deaths occurred in the ^131^I-LNTH-1095 + enzalutamide group and 17 (42.5%) deaths in the enzalutamide monotherapy group. There was no significant decrease in the risk of death between the groups [hazard ratio (HR) = 1.10; 95% CI, 0.62–1.96; *P* =0.59; Fig. 3B]. The median follow-up time was 15.9 months (range: 0.1–41.5), and the median time to death was 18.8 months (95% CI, 15.34–29.47) in the ^131^I-LNTH-1095 + enzalutamide group and 22 months [95% CI, 14.59–not estimable (NE)] in enzalutamide monotherapy group. At the median follow-up, the median OS had not been reached, with fewer than 50% of events observed in both arms (40% for ^131^I-LNTH-1095 + enzalutamide vs. 34% for enzalutamide monotherapy).

#### Time to PSA progression

PSA progression occurred in 35 (43.8%) subjects in the ^131^I-LNTH-1095 + enzalutamide group and in 21 (52.5%) subjects in the enzalutamide monotherapy group. Due to less than 50% of the combination therapy group experiencing progression, the median time to PSA progression was NE, compared with 10 months (95% CI, 3.5 months to NE) in the monotherapy group. No significant differences were observed between treatment groups within either risk stratum (*P* = 0.67 for intermediate-risk and *P* = 0.79 for high-risk prostate cancer).

#### DoR

In total, seven subjects from the ^131^I-LNTH-1095 + enzalutamide group and six from the enzalutamide monotherapy group were assessed by the local investigator as having a PR or CR and thus were included in the DoR analysis. The median DoR was similar between the treatment groups: 15 months (95% CI, 7.52 months to NE) in the ^131^I-LNTH-1095 + enzalutamide group versus 15.1 months (95% CI, 3.3 months to NE) in the enzalutamide monotherapy group (*P* = 0.63).

#### Time to next treatment

A total of 35 (43.8%) subjects in the ^131^I-LNTH-1095 + enzalutamide group and 28 subjects in the enzalutamide monotherapy group started a new anticancer treatment. The median time to next treatment was statistically longer in the combination therapy group (18.3 months) compared with the enzalutamide monotherapy group (10.9 months; *P* = 0.001; Supplementary Fig. S1). This difference was significant in the analysis of intermediate-risk (median time to event: 18.3 vs. 12.3 months, *P* = 0.02) and high-risk (median time to event: NE vs. 5.9 months, *P* = 0.0001) groups.

### Exploratory efficacy endpoints

#### ^18^F-piflufolastat uptake

Mean (SE) baseline SUV_max_ was 52.2 (5.2) in the ^131^I-LNTH-1095 + enzalutamide group and 50.3 (5) in the enzalutamide-only group. At week 53 (EOT), median (range) change from baseline was −15.7 (−178.4 to 61.4) in the ^131^I-LNTH-1095 + enzalutamide group and 1.58 (−101.4 to 178.6) in the enzalutamide-only group (Table 3). The least-squares mean (LSM) of difference between treatment groups was −30.1 (11.2; 95% CI, −52.3 to −8). The difference between treatment groups was statistically significant (*P* = 0.003).

**Table 3.** Summary of ^18^F-piflufolastat maximum standardized uptake value (SUV_max_) at baseline and EOT.

Maximum SUV overall	^131^I-LNTH-1095 + enzalutamide (*N* = 80)	Enzalutamide monotherapy (*N* = 40)	Treatment effect
Baseline	​	​	​
*N*	69	35	​
Mean (SE)	52.22 (5.238)	50.31 (5.002)	​
Median (min, max)	38.34 (4.8, 214.3)	45.32 (7.2, 115.5)	​
EOT week 53^a^	​	​	​
*n*	42	21	​
Mean (SE)	28.29 (4.358)	60.47 (13.441)	​
Median (min, max)	22.04 (1, 130.2)	36 (13.5, 263.3)	​
CFB: mean (SE)	−32.11 (7.264)	5.70 (13.345)	​
CFB: median (min, max)	−15.67 (−178.4, 61.4)	1.58 (−101.4, 178.6)	​
CFB: LS mean (SE)	−27.9 (6.4)	2.3 (9.2)	​
CFB: 95% CI for LS mean	(−40.6 to −15.2)	(−15.9 to 20.4)	​
LSM difference (SE) from control	​	​	−30.1 (11.2)
95% CI for LSM difference	​	​	(−52.3 to −8)
*P* value^b^	​	​	0.008
Overall effect	​	​	​
*P* value for treatment	​	​	0.003

Abbreviations: ANOVA, analysis of variance; CFB, change from baseline; LS, least squares.

a Subjects who discontinued treatment prior to study completion were not available for the EOT week 53 timepoint.

b From a repeated-measures ANOVA with parameters treatment, visit, and treatment-by-visit interaction as factors. Missing data are assumed to be missing at random, and no imputation of missing values is performed.

When SUV_max_ was compared between confirmed PSA_50_ responders versus nonresponders, both treatment groups had greater changes in median (range) from baseline in the responders compared with nonresponders [enzalutamide-only: −7.78 (−101.38 to 28.96) vs. 9.92 (−22.85 to 178.6); ^131^I-LNTH-1095 + enzalutamide: −25.24 (−178.36 to 61.4) vs. −2.27 (−16.02 to 11.22); Supplementary Tables S7 and S8]. Additionally, stratification by the number of ^131^I-LNTH-1095 therapy cycles resulted in similar decreases in mean and median SUV_max_ across cycles (Supplementary Table S9).

#### Time from randomization to first symptomatic skeletal event (SSE)

Six (7.5%) subjects in the ^131^I-LNTH-1095 + enzalutamide group and 5 (12.5%) subjects in the enzalutamide-only group had an SSE during the study. The event-free rate at 12 months was 0.9 (95% CI, 0.81–0.96) in the ^131^I-LNTH-1095 + enzalutamide group and 0.9 (95% CI, 0.71–0.94) in the enzalutamide-only group. Because less than half of the subjects had an SSE, median time to first SSE could not be calculated.

#### Progression in pain intensity (BPI-SF)

At 6 months, 2 (95% CI, 0–9) subjects in the ^131^I-LNTH-1095 + enzalutamide group and 3 (95% CI, 2–21) subjects in the enzalutamide-only group had pain progression. The difference in the proportion between the treatment groups was −5.19 (95% CI, −19 to 4) and was not statistically significant (*P* = 0.334).

#### FACT-P questionnaire score

The mean (SE) baseline FACT-P overall score was 93.40 (2.72) in the ^131^I-LNTH-1095 + enzalutamide group and 89.17 (2.62) in the enzalutamide-only group. At week 53 (EOT), LSM change from baseline was −3.3 (95% CI, −8.9 to 2.2) in the ^131^I-LNTH-1095 + enzalutamide group and −3.3 (95% CI, −8.2 to 1.7) in the enzalutamide-only group, with LSM difference from control of −0.1 (95% CI, −7.5 to 7.4). The overall treatment effect was not statistically significant (*P* = 0.630).

#### SF-12 component scores

The mean (SE) baseline SF-12 Mental Component Score was 51.13 (1.13) in the ^131^I-LNTH-1095 + enzalutamide group and 50.91 (1.58) in the enzalutamide-only group. At week 53 (EOT), the LSM change from baseline was −3.4 (95% CI, −6.9 to 0) in the ^131^I-LNTH-1095 + enzalutamide group and 0.6 (95% CI, −2.7 to 3.9) in the enzalutamide-only group, with LSM difference from control of −4 (95% CI, −8.8 to 0.8). The overall treatment effect was not statistically significant (*P* = 0.175).

The mean (SE) baseline SF-12 Physical Component Summary score was 43.02 (2.13) in the ^131^I-LNTH-1095 + enzalutamide group and 37.65 (2.62) in the enzalutamide-only group. At week 53 (EOT), LSM change from baseline was −5.6 (95% CI, −9 to −2.1) for the ^131^I-LNTH-1095 + enzalutamide group and −5.8 (95% CI, −9.2 to −2.5) for the enzalutamide-only group, with LSM difference from control of 0.3 (95% CI, −4.6 to 5.1). The overall treatment effect was not statistically significant (*P* = 0.866).

#### EQ-5D-5L and EuroQol VAS

The difference between treatment groups in EQ VAS was not statistically significant (*P* = 0.517) at any visit. The mean (SE) baseline EQ-5D-5L VAS score was 74.4 (2.69) in the ^131^I-LNTH-1095 + enzalutamide group and 69.9 (4.21) in the enzalutamide-only group. At week 53 (EOT), LSM change from baseline was −0.9 (95% CI, −8.4 to 6.7) in the ^131^I-LNTH-1095 + enzalutamide arm and −1.3 (95% CI, −9 to 6.4) in the enzalutamide-only arm, with LSM difference between treatment groups of 0.5 (95% CI, −10.3 to 11.3).

#### aBSI

The mean (SE) baseline aBSI was 0.019 (0.003) in the ^131^I-LNTH-1095 + enzalutamide group and 0.014 (0.004) in the enzalutamide-only group. At week 36, LSM change from baseline was −0.006 (95% CI, −0.015 to 0.002) in the ^131^I-LNTH-1095 + enzalutamide group and 0.003 (95% CI, −0.008 to 0.014) in the enzalutamide-only group, with LSM difference from control of −0.009 (95% CI, −0.024 to 0.005). The overall treatment effect was not statistically significant (*P* = 0.187).

#### ECOG performance status

The mean (SE) baseline ECOG performance score 0.4 (0.06) in the ^131^I-LNTH-1095 + enzalutamide group and 0.3 (0.09) in the enzalutamide-only group. At week 53 (EOT), the LSM change from baseline was 0.6 (95% CI, 0.4–0.8) in the ^131^I-LNTH-1095 + enzalutamide group and 0.4 (95% CI, 0.1–0.6) in the enzalutamide-only group, with LSM difference from control of 0.3 (95% CI: −0.1 to 0.6). The overall treatment effect was not statistically significant (*P* = 0.128).

#### Safety

Supplementary Table S10 lists common TEAEs, including those of grade ≥3 severity, in subjects treated with ^131^I-LNTH-1095 + enzalutamide and enzalutamide monotherapy. Most subjects (95.7%; 110/115) had ≥1 TEAE during the study. The most common TEAEs in the ^131^I-LNTH-1095 + enzalutamide group were fatigue/asthenia (75%), nausea (59.2%), thrombocytopenia (51.3%), decreased appetite (48.7%), and dry mouth (47.4%).

Bone marrow effects were more common among the subjects who received ^131^I-LNTH-1095 + enzalutamide compared with those who received enzalutamide monotherapy: platelet count decreased/thrombocytopenia (51.3% vs. 0%), lymphocyte count decreased/lymphopenia (22.4% vs. 5.1%), neutrophil count decreased/neutropenia (19.7% vs. 2.6%), and hemoglobin decreased/anemia (42.1% vs. 15.4%). Xerostomia (grades 1 and 2 only) was more common in subjects treated with ^131^I-LNTH-1095 + enzalutamide than in those who received enzalutamide monotherapy: 47.4% versus 2.6%.

Grade ≥3 TEAEs were reported in 66 (57.4%) subjects: 65.8% of the ^131^I-LNTH-1095 + enzalutamide group versus 41% of the enzalutamide monotherapy group. Of the 213 (total, nonsubject level) grade ≥3 TEAEs, 7 were fatal (for 3 subjects), 63 were not recorded as recovered by the end of study, and 143 had resolved. Of those TEAEs that recovered, fatigue/asthenia (4 events) had recovery times that ranged from 6 to 52 days; nausea (1 event) resolved in 4 days; and thrombocytopenia (28 resolved events) had recovery times that ranged from 1 to 215 days, with a median of 19.5 days. Anorexia (1 event) did not report a time to recovery.

Serious TEAEs (SAE) were reported in 29 (38.2%) subjects in the ^131^I-LNTH-1095 + enzalutamide group and 11 (28.2%) in the enzalutamide monotherapy group. The most common SAEs for each treatment group are presented in Supplementary Table S11.

Most SAEs were judged unrelated to study drug. In the combination group, 81.6% (62/76) had ≥1 TEAE related to ^131^I-LNTH-1095. A similar proportion of subjects in the combination and monotherapy groups experienced enzalutamide-related TEAEs (63.2% vs. 66.7%). No SAEs related to ^18^F-piflufolastat were reported.

In the ^131^I-LNTH-1095 + enzalutamide group, 8 (10.5%) subjects had TEAEs that led to discontinuation of either agent and 1 (1.3%) subject discontinued both. In the enzalutamide-only group, 2 (5.1%) subjects discontinued enzalutamide because of TEAEs.

Four deaths due to TEAEs occurred: 3 (3.9%) in the ^131^I-LNTH-1095 + enzalutamide group (one subject with pancytopenia; one with dehydration, acute kidney injury, septic shock, and pancytopenia; and one with anemia) and 1 (2.6%) in the enzalutamide monotherapy group (subdural hematoma). Two deaths in the ^131^I-LNTH-1095 + enzalutamide group were considered treatment-related. The third death also involved additional events (a fall resulting in a subdural hematoma approximately 1.5 months prior to death) deemed unrelated to the combination therapy. The death in the monotherapy group was unrelated to enzalutamide.

## Discussion

The ARROW study met its primary endpoint, showing significantly improved PSA_50_ response in mCRPC subjects treated with ^131^I-LNTH-1095 + enzalutamide after progression on abiraterone compared with enzalutamide alone. This study demonstrated LNTH-1095 to be an effective and tolerable PSMA-targeting small molecule when combined with an ARPI for treating mCRPC. It should be noted that the median time from diagnosis to study treatment differed between the two treatment groups, possibly due to the higher proportion of subjects in the enzalutamide monotherapy arm who initially presented with synchronous metastatic disease, potentially affecting the observed outcomes.

In addition to ^131^I-LNTH-1095, ^177^Lu-PSMA-617 and ^177^Lu-PSMA-I&T are PSMA-targeted small-molecule radioligands under investigation for the treatment of advanced prostate cancer (23–26). ^177^Lu-PSMA-617–positive results from the VISION trial (NCT03511664) and FDA approval marked a paradigm shift in mCRPC management after ARPI and taxane chemotherapy (3). Similar to this study, the ENZA-p trial (NCT04419402) examined ^177^Lu-PSMA-617 + enzalutamide versus enzalutamide alone in pre-chemotherapy patients with mCRPC, demonstrating significant improvements in PSA PFS (13 vs. 7.8 months) and OS (34 vs. 26 months; refs. 27, 28). Although the dosing schedule was different: 3.7 GBq (100 mCi) × 4, 8–week time interval for ARROW versus an adaptive regimen of 7.4 GBq (≈200 mCi), 6 to 8 week time interval (2–4 doses based on interim PSMA PET/CT), given the differences in half-life and emission energies between ^131^I and ^177^Lu, comparisons based solely on administered activity may not accurately reflect differences in radiation dose distribution and therapeutic efficacy.

Both ARROW and ENZA-p underscore the benefits of combination therapy. Currently, the PSMACare (NCT05849298) and PSMAndARPI (NCT06894511) trials compare the efficacy and safety of ^177^Lu-PSMA-617 alone versus in combination with ARPIs in similar patient populations (first-line mCRPC; refs. 29, 30). In the metastatic hormone-sensitive prostate cancer (mHSPC) setting, the UpFrontPSMA trial (NCT04343885) demonstrated promising results with the sequential administration of ^177^Lu-PSMA-617 followed by docetaxel, compared with docetaxel alone, supporting the potential role of PSMA RLT earlier in the disease course (31).

The ^131^I-LNTH-1095 + enzalutamide safety profile was acceptable, with 10.5% of subjects discontinuing because of TEAEs. Grade ≥3 AEs were more frequent with combination therapy, particularly hematologic toxicity. Bone marrow effects were observed with ^131^I-LNTH-1095 + enzalutamide, particularly among subjects with a high bone tumor burden. In addition, the incidence of hematologic TEAEs such as anemia (42.1%) and thrombocytopenia (51.3%) were more frequent when compared with other PSMA RLT studies such as ENZA-p [anemia (10%) and thrombocytopenia (0%)] and PSMAfore [anemia (27%) and thrombocytopenia (7%); refs. 24, 27]. Three of 76 (3.9%) subjects who received ^131^I-LNTH-1095 + enzalutamide died from TEAEs, and two (2.6%) were deemed treatment-related. Prior radiotherapy and/or prostate cancer bone marrow metastases may have influenced hematologic AEs and should be considered in future studies. These findings also call into question the potential benefit of using ^131^I over ^177^Lu in this patient population.

^177^Lu emits moderate-energy γ rays [208 keV (11%) and 113 keV (7%)], adequate for imaging with minimal radiation exposure (32), whereas ^131^I’s higher γ emission [364 keV (82%) and 637 keV (6.5%)] may contribute to DNA damage via reactive oxygen species, which can induce apoptosis and other cellular effects (33–36). Whether these mechanistic differences may have therapeutic relevance in patient selection remains unclear. However, in this analysis, protocol-defined high-risk mCRPC subjects had a greater improvement in rPFS with ^131^I-LNTH-1095 + enzalutamide, emphasizing a potentially greater role for this combination under further optimized conditions in subjects who may have higher volumes of disease and worse prognosis.

The ARROW study, while providing valuable insights, has several limitations. First, it was not powered to detect differences in rPFS and OS, limiting conclusions drawn from the study. Second, the injected dose activity (3.7 GBq ×4/8 weeks) was half that used in the other main ^177^Lu-PSMA RLT trials (VISION and PSMAfore: 7.4 GBq ×6/6 weeks; SPLASH: 6.8 GBq ×4/8 weeks). Furthermore, ^131^I as a targeted radioisotope presents unique challenges, including γ emissions that necessitate radioprotection measures for staff and family, an increased risk of thyroid abnormalities (37), and competition with ^177^Lu, a widely adopted short-range β-emitter for prostate cancer. Finally, the use of ^131^I-LNTH-1095 may be constrained by specialized facility and personnel requirements, increasing costs, and limiting access. Future development plans will require reevaluating the optimal radioisotope for potential therapeutic use with this PSMA-targeted ligand.

### Conclusion

The ARROW study demonstrated the clinical efficacy of ^131^I-LNTH-1095 in combination with enzalutamide despite a lower dosing schedule (4 cycles of 3.7 GBq/dose every 8 weeks) than the other approved PSMA RLT agents, with a safety profile consistent with known RLT effects. Additional studies may be warranted to evaluate long-term outcomes in chemo-naïve mCRPC, provided they are within the context of a non-^131^I radioisotope and a further optimized dosing regimen.

## Supplementary Material

Supplementary Table S1 Summary of Subject Disposition (All subjects)

Supplementary Table S2 Demographic and Baseline characteristics of the Safety Population

Supplementary Table S3 Baseline Tumor Characteristics of the Safety Population

Supplementary Table S4 Prostate Cancer History

Supplementary Table S5 Representativeness of Study Participants

Supplementary Table S6 Subgroup Analyses of PSA50 Response

Supplementary Table S7 Summary of 18F-piflufolastat SUVmax in PSA50 Responders vs. Non-responders Enzalutamide

Supplementary Table S8 Summary of 18F-piflufolastat SUVmax in PSA50 Responders vs Non-responders Combination

Supplementary Table S9 Summary of 18F-piflufolastat SUVmax by Number of 131I-LNTH-1095 Therapy Cycles

Supplementary Table S10 Treatment-Emergent Adverse Events

Supplementary Table S11 Most Common Serious Treatment-Emergent Adverse Events

Supplementary Figure S1 Time to First New Anti-Cancer Treatment

## Data Availability

Most of the data generated in this study are available within the article and its Supplementary Data files. For all additional raw data, a community-recognized, structured repository does not exist, but these data are available upon reasonable request from the corresponding author.
