# Supplementary material for: 131I-LNTH-1095 Radioligand Therapy plus Enzalutamide versus Enzalutamide Alone in Men with PSMA-Avid Metastatic Castration-Resistant Prostate Cancer: A Phase II Study
Source: Clin Cancer Res. 2026 Mar 4;32(10):1973–82. doi: 10.1158/1078-0432.CCR-25-4948 (PMC13176818; doi:10.1158/1078-0432.CCR-25-4948)
Supplement: Supplementary Table S1 — Summary of Subject Disposition (All subjects) [file ccr-25-4948_supplementary_table_s1_suppts1.docx]

**Supplementary Table S1. Summary of Subject Disposition (All subjects)**

|  | ^131^I-LNTH-1095+ enzalutamide n (%) | Enzalutamide monotherapy n (%) | All subjects n (%) |
| --- | --- | --- | --- |
| Subjects screened |  |  | 177 |
| Any screen failures ^a^ |  |  | 57 (32.2) |
| PSMA Imaged Set |  |  | 132 |
| Met PSMA avidity |  |  | 123 (93.2) |
| Did not meet PSMA avidity |  |  | 6 (4.5) |
| Missing |  |  | 3 (2.3) |
| Randomized (Full Analysis Set) ^b,c^ | 80 | 40 | 120 |
| Stratification ^d^ |  |  |  |
| Intermediate-risk prostate cancer | 63 (78.8) | 31 (77.5) | 94 (78.3) |
| High-risk prostate cancer | 17 (21.3) | 9 (22.5) | 26 (21.7) |
| Safety set | 76 (95.0) | 39 (97.5) | 115 (95.8) |
| Completed randomization treatment period | 29 (36.3) | 13 (32.5) | 42 (35.0) |
| Subjects discontinued randomized treatment | 47 (58.8) | 26 (65.0) | 73 (60.8) |
| Radiographic progression | 17 (21.3) | 14 (35.0) | 31 (25.8) |
| Treatment is no longer benefiting subject | 6 (7.5) | 5 (12.5) | 11 (9.2) |
| Adverse event | 8 (10.0) | 1 (2.5) | 9 (7.5) |
| Unequivocal clinical progression | 5 (6.3) | 2 (5.0) | 7 (5.8) |
| Other | 4 (5.0) | 0 (0.0) | 4 (3.3) |
| Death | 3 (3.8) | 0 (0.0) | 3 (2.5) |
| Subject decision | 3 (3.8) | 0 (0.0) | 3 (2.5) |
| Withdrawal of consent | 1 (1.3) | 3 (7.5) | 4 (3.3) |
| Investigator decision | 0 (0.0) | 1 (2.5) | 1 (0.8) |
| The PSMA Imaged Population is all subjects who received any dose of ^18^F-piflufolastat. The Safety Set is all subjects who received any dose of study drug (^131^I-LNTH-1095 or enzalutamide). The full analysis set was defined as all subjects assigned to a treatment group per the randomization schedule.  PSA = prostate-specific antigen; PSMA = prostate-specific membrane antigen  ^a^ Screen failures included subjects who failed to meet all inclusion/exclusion criteria and subjects who were not PSMA-avid.  ^b^  Four subjects met PSMA-avidity but were not randomized, and 1 subject was not PSMA imaged but was randomized.  ^c^ Two subjects were randomized and later found to be screen failures.  ^d^  Stratification risk classes:  • Intermediate-risk prostate cancer: hemoglobin ≥11 g/dL, lactate dehydrogenase <262 IU/L, and alkaline phosphatase <414 IU/L  • High-risk prostate cancer: hemoglobin <11 g/dL, or lactate dehydrogenase ≥262 IU/L, or alkaline phosphatase ≥ 414 IU/L | | | |
