# Supplementary material for: 131I-LNTH-1095 Radioligand Therapy plus Enzalutamide versus Enzalutamide Alone in Men with PSMA-Avid Metastatic Castration-Resistant Prostate Cancer: A Phase II Study
Source: Clin Cancer Res. 2026 Mar 4;32(10):1973–82. doi: 10.1158/1078-0432.CCR-25-4948 (PMC13176818; doi:10.1158/1078-0432.CCR-25-4948)
Supplement: Supplementary Table S2 — Demographic and Baseline characteristics of the Safety Population [file ccr-25-4948_supplementary_table_s2_suppts2.docx]

**Supplementary Table S2. Demographic and Baseline characteristics of the Safety Population**

|  | ^131^I-LNTH-1095+ enzalutamide (N=76) | Enzalutamide (N=39) | All Subjects (N=115) |
| --- | --- | --- | --- |
| Age, years |  |  |  |
| N | 76 | 39 | 115 |
| Mean (SD) | 70.9 (8.96) | 70.2 (8.97) | 70.7 (8.93) |
| Median | 72.0 | 70.0 | 72.0 |
| Min, Max | 49, 86 | 52, 86 | 49, 86 |
| Age category, n (%) |  |  |  |
| <65 | 19 (25.0) | 11 (28.2) | 30 (26.1) |
| 65 to <75 | 25 (32.9) | 18 (46.2) | 43 (37.4) |
| ≥75 | 32 (42.1) | 10 (25.6) | 42 (36.5) |
| Sex |  |  |  |
| Male, n | 76 | 39 | 115 |
| Ethnicity, n (%) |  |  |  |
| Hispanic or Latino | 3 (3.9) | 2 (5.1) | 5 (4.3) |
| Not Hispanic or Latino | 71 (93.4) | 36 (92.3) | 107 (93.0) |
| Not Reported | 2 (2.6) | 1 (2.6) | 3 (2.6) |
| Race, n (%) |  |  |  |
| Asian | 2 (2.6) | 0 (0.0) | 2 (1.7) |
| Black or African American | 5 (6.6) | 6 (15.4) | 11 (9.6) |
| White | 64 (84.2) | 31 (79.5) | 95 (82.6) |
| Multiple | 1 (1.3) | 1 (2.6) | 2 (1.7) |
| Other | 2 (2.6) | 0 (0.0) | 2 (1.7) |
| Not Reported or Unknown | 2 (2.6) | 1 (2.6) | 3 (2.6) |
| Height at Baseline, cm |  |  |  |
| N | 73 | 37 | 110 |
| Mean (SD) | 174.7 (7.53) | 174.3 (6.52) | 174.6 (7.18) |
| Median | 175.0 | 175.3 | 175.0 |
| Min, Max | 157, 191 | 158, 186 | 157, 191 |
| Weight at Baseline, kg |  |  |  |
| N | 73 | 37 | 110 |
| Mean (SD) | 90.58 (17.467) | 90.77 (19.280) | 90.64 (18.008) |
| Median | 90.60 | 85.30 | 89.20 |
| Min, Max | 59.9, 152.0 | 56.0, 150.9 | 56.0, 152.0 |
| BMI at Baseline, kg/m^2^ |  |  |  |
| N | 72 | 37 | 109 |
| Mean (SD) | 29.66 (4.629) | 29.73 (5.350) | 29.68 (4.861) |
| Median | 29.50 | 29.30 | 29.30 |
| Min, Max | 21.7, 42.6 | 21.4, 46.6 | 21.4, 46.6 |

The Safety population is defined as all subjects who received any dose of study drug, defined as ^18^F-piflufolastat, ^131^I-LNTH-1095, or enzalutamide. All subjects are assigned to the treatment to which they were randomized. Age is calculated as the number of years between the date of birth and the date of signing of the informed consent form.

BMI = body mass index; Max = maximum; Min = minimum; SD = standard deviation.
