# Supplementary material for: 131I-LNTH-1095 Radioligand Therapy plus Enzalutamide versus Enzalutamide Alone in Men with PSMA-Avid Metastatic Castration-Resistant Prostate Cancer: A Phase II Study
Source: Clin Cancer Res. 2026 Mar 4;32(10):1973–82. doi: 10.1158/1078-0432.CCR-25-4948 (PMC13176818; doi:10.1158/1078-0432.CCR-25-4948)
Supplement: Supplementary Table S3 — Baseline Tumor Characteristics of the Safety Population [file ccr-25-4948_supplementary_table_s3_suppts3.docx]

**Supplementary Table S3. Baseline Tumor Characteristics of the Safety Population**

|  | ^131^I-LNTH-1095+enzalutamide (N=76) | Enzalutamide (N=39) | All Subjects (N=115) |
| --- | --- | --- | --- |
| Primary tumor (T) stage at diagnosis, n (%) |  |  |  |
| T1 – not visible on imaging | 14 (18.4) | 4 (10.3) | 18 (15.7) |
| T2 – confined within prostate | 23 (30.3) | 12 (30.8) | 35 (30.4) |
| T3 – extends through prostate capsule | 20 (26.3) | 12 (30.8) | 32 (27.8) |
| T4 – fixed/invades adjacent structures | 10 (13.2) | 9 (23.1) | 19 (16.5) |
| Regional lymph node (N) stage, n (%) |  |  |  |
| NX – not assessed | 13 (17.1) | 5 (12.8) | 18 (15.7) |
| N0 – no metastasis | 29 (38.2) | 13 (33.3) | 42 (36.5) |
| N1 – metastasis | 32 (42.1) | 20 (51.3) | 52 (45.2) |
| Distant metastasis (M) stage, n (%) |  |  |  |
| MX – not assessed | 12 (15.8) | 5 (12.8) | 17 (14.8) |
| M0 – no distant metastasis | 32 (42.1) | 12 (30.8) | 44 (38.3) |
| M1 – distant metastasis | 31 (40.8) | 21 (53.9) | 52 (45.2) |
| M1a – non-regional lymph node | 1 (1.3) | 1 (2.6) | 2 (1.7) |
| M1b – bones | 23 (30.3) | 18 (46.2) | 41 (35.7) |
| M1c – other site(s) | 7 (9.2) | 2 (5.1) | 9 (7.8) |
| Gleason Score, n (%) |  |  |  |
| 6 | 5 (6.6) | 1 (2.6) | 6 (5.2) |
| 7 | 18 (23.7) | 5 (12.8) | 23 (20.0) |
| 8 | 10 (13.2) | 7 (17.9) | 17 (14.8) |
| 9 | 32 (42.1) | 17 (43.6) | 49 (42.6) |
| 10 | 6 (7.9) | 3 (7.7) | 9 (7.8) |
|  | | | |

Staging data presented in this table reflect disease status at initial diagnosis, rather than at trial enrollment. M1a/b/c categories were assigned based on the highest-risk metastatic site present at baseline.
