# Supplementary material for: 131I-LNTH-1095 Radioligand Therapy plus Enzalutamide versus Enzalutamide Alone in Men with PSMA-Avid Metastatic Castration-Resistant Prostate Cancer: A Phase II Study
Source: Clin Cancer Res. 2026 Mar 4;32(10):1973–82. doi: 10.1158/1078-0432.CCR-25-4948 (PMC13176818; doi:10.1158/1078-0432.CCR-25-4948)
Supplement: Supplementary Table S4 — Prostate Cancer History [file ccr-25-4948_supplementary_table_s4_suppts4.docx]

**Supplementary Table S4. Prostate Cancer History**

|  | ^131^I-LNTH-1095+enzalutamide (N=76) | Enzalutamide (N=39) | All Subjects (N=115) |
| --- | --- | --- | --- |
| Months since initial prostate cancer pathologic diagnosis ^a^ |  |  |  |
| n | 71 | 36 | 107 |
| Mean (SD) | 76.7 (62.19) | 64.3 (57.15) | 72.5 (60.55) |
| Median | 63.8 | 38.3 | 54.5 |
| Months since pathologic confirmed metastatic disease |  |  |  |
| n | 45 | 22 | 67 |
| Mean (SD) | 48.57 (40.347) | 41.80 (42.273) | 46.35 (40.794) |
| Median | 35.12 | 27.14 | 32.62 |
| Baseline PSA (ng/mL) |  |  |  |
| N | 74 | 37 | 111 |
| Mean (SD) | 39.80 (63.792) | 48.73 (113.974) | 42.78 (83.485) |
| Median | 14.46 | 10.88 | 11.70 |

^a^Months since initial diagnosis is calculated as the number of months between the date of diagnosis or last surgery and the date of signing the informed consent.
