# Supplementary material for: 131I-LNTH-1095 Radioligand Therapy plus Enzalutamide versus Enzalutamide Alone in Men with PSMA-Avid Metastatic Castration-Resistant Prostate Cancer: A Phase II Study
Source: Clin Cancer Res. 2026 Mar 4;32(10):1973–82. doi: 10.1158/1078-0432.CCR-25-4948 (PMC13176818; doi:10.1158/1078-0432.CCR-25-4948)
Supplement: Supplementary Table S5 — Representativeness of Study Participants [file ccr-25-4948_supplementary_table_s5_suppts5.docx]

| **Supplementary Table S5. Representativeness of Study Participants*** | |
| --- | --- |
| Cancer type(s)/subtype(s)/stage(s)/condition | Metastatic Castration-Resistant Prostate Cancer (mCRPC) |
| Considerations related to: | |
| Sex | Prostate cancer occurs only in men. |
| Age | Prostate cancer is more common in older men than younger men. The median age at diagnosis (2018-2022) is 68 years old. Incidence rates are highest per 100,00 at 70-74 years of age (759.4). |
| Race/ethnicity | SEER 5-Year age-adjusted incidence rates per 100,000 (2018-2022) are: Hispanic (any race): 90.2; Non-Hispanic American Indian/Alaska Native: 82.2; Non-Hispanic Asian/Pacific Islander: 64.5; Non-Hispanic Black: 194.8; Non-Hispanic White: 118.9 ; Black (includes Hispanic): 187.6; White (includes Hispanic): 112.5. |
| Geography | In the US, the rate of new cases of prostate cancer was 120.2 per 100,000 men per year. The death rate was 19.2 per 100,000 men per year. These rates are age-adjusted and based on 2018–2022 cases and 2019–2023 deaths. The lifetime risk of developing cancer in the US is ~12.9%, based on 2018–2021 data, excluding 2020 due to COVID. In 2022, there were an estimated 3,518,978 men living with prostate cancer in the United States. |
| Other considerations | The SEER age-adjusted incidence rates per 100,000 men for rural and urban areas (2022) were 115.0 (rural) and 125.7 (urban) |
| Overall representativeness of this study | Treatment arms were demographically and clinically balanced and consistent with the literature. In the safety set (n=115), 82.6% were white, 9.6% Black/African American, 1.7% Asian, and 4.3% Hispanic/Latino. Baseline characteristics, including tumor burden and disease history, are summarized in Supplementary Tables S2-S4. |
| *all non-study data are derived from the (U.S. only) SEER database: https://seer.cancer.gov/statfacts/html/prost.html; Canadian data are not readily available for race/ethnicity. | |
