# Supplementary material for: 131I-LNTH-1095 Radioligand Therapy plus Enzalutamide versus Enzalutamide Alone in Men with PSMA-Avid Metastatic Castration-Resistant Prostate Cancer: A Phase II Study
Source: Clin Cancer Res. 2026 Mar 4;32(10):1973–82. doi: 10.1158/1078-0432.CCR-25-4948 (PMC13176818; doi:10.1158/1078-0432.CCR-25-4948)
Supplement: Supplementary Table S6 — Subgroup Analyses of PSA50 Response [file ccr-25-4948_supplementary_table_s6_suppts6.docx]

**Supplementary Table S6. Subgroup Analyses of PSA_50_ Response**

|  | **^131^I-LNTH-1095+enzalutamide** | **Enzalutamide** |
| --- | --- | --- |
| **Age Group: 65 to <75 years, N** | 27 | 19 |
| Evaluable cases, n | 21 | 14 |
| ≥50% Decline in PSA, n (%) | 14 (66.7) | 2 (14.3) |
| 95% CI^1^ | 43.0–85.4] | 1.8–42.8 |
| *P*-value^2^ | 0.0027 | |
|  |  | |
| **Race Group: White, N** | 68 | 32 |
| Evaluable cases, n | 59 | 26 |
| ≥50% Decline in PSA, n (%) | 38 (64.4) | 7 (26.9) |
| 95% CI^1^ | 50.9–76.4 | 11.6–47.8 |
| *P*-value^2^ | 0.0015 | |
|  |  | |
| **Ethnicity: Not Hispanic or Latino, N** | 75 | 36 |
| Evaluable cases, n | 65 | 30 |
| ≥50% Decline in PSA, n (%) | 41 (63.1) | 9 (30.0) |
| 95% CI^1^ | 50.2–74.7 | 14.7–49.4 |
| *P*-value^2^ | 0.0028 | |
|  |  | |
| **LDH Subgroup: ≤ ULN, N** | 56 | 28 |
| Evaluable cases, n | 51 | 25 |
| ≥50% Decline in PSA, n (%) | 35 (68.6) | 9 (36.0) |
| 95% CI^1^ | 54.1–80.9 | 18.0–57.5 |
| *P*-value^2^ | 0.0072 | |

CI = confidence interval; PSA = prostate specific antigen; ULN = upper limit of normal

^1^Exact 95% confidence intervals are estimated based on the binomial distribution.

^2^The P-value is based on the Cochran-Mantel-Haenszel chi-square statistic of the ‘row mean score differ’ alternative hypothesis.
