# Supplementary material for: 131I-LNTH-1095 Radioligand Therapy plus Enzalutamide versus Enzalutamide Alone in Men with PSMA-Avid Metastatic Castration-Resistant Prostate Cancer: A Phase II Study
Source: Clin Cancer Res. 2026 Mar 4;32(10):1973–82. doi: 10.1158/1078-0432.CCR-25-4948 (PMC13176818; doi:10.1158/1078-0432.CCR-25-4948)
Supplement: Supplementary Table S7 — Summary of 18F-piflufolastat SUVmax in PSA50 Responders vs. Non-responders Enzalutamide [file ccr-25-4948_supplementary_table_s7_suppts7.docx]

**Supplementary Table S7. Summary of ^18^F-piflufolastat Maximum Standardized Uptake Value (SUV_max_) in PSA_50_ Responder vs. Non-responder at Baseline and End of Treatment: Enzalutamide Monotherapy Group**

| **Maximum SUV Overall** | **Enzalutamide** | |
| --- | --- | --- |
|  | **Responder** | **Non-responder** |
|  |  |  |
| **Baseline** |  |  |
| n | 8 | 11 |
| Mean (SE) | 50.30 (13.920) | 60.76 (8.771) |
| Median (Min, Max) | 39.04 (10.33, 115.48) | 50.43 (20.38, 99.75) |
|  |  |  |
| **EOT Week 53** |  |  |
| n | 8 | 11 |
| Mean (SE) | 26.67 (7.891) | 87.79 (22.230) |
| Median (Min, Max) | 16.42 (13.54, 79.12) | 61.01 (25.05, 263.27) |
| CFB: Mean (SE) | -23.62 (16.391) | 27.02 (17.576) |
| CFB: Median (Min, Max) | -7.78 (-101.38, 28.96) | 9.92 (-22.85, 178.6) |

CFB = Change from Baseline; SE=Standard Error; CI=Confidence Interval.

Missing data are assumed to be missing at random, and no imputation of missing values is performed.
