# Supplementary material for: 131I-LNTH-1095 Radioligand Therapy plus Enzalutamide versus Enzalutamide Alone in Men with PSMA-Avid Metastatic Castration-Resistant Prostate Cancer: A Phase II Study
Source: Clin Cancer Res. 2026 Mar 4;32(10):1973–82. doi: 10.1158/1078-0432.CCR-25-4948 (PMC13176818; doi:10.1158/1078-0432.CCR-25-4948)
Supplement: Supplementary Table S8 — Summary of 18F-piflufolastat SUVmax in PSA50 Responders vs Non-responders Combination [file ccr-25-4948_supplementary_table_s8_suppts8.docx]

**Supplementary Table S8. Summary of ^18^F-piflufolastat Maximum Standardized Uptake Value (SUV_max_) in PSA50 Responders vs. Non-responders at Baseline and End of Treatment: Combination Therapy Group**

| **Maximum SUV Overall** | **^131^I-LNTH-1095+ Enzalutamide** | |
| --- | --- | --- |
|  | **Responders** | **Non-responders** |
|  |  |  |
| **Baseline** |  |  |
| n | 36 | 5 |
| Mean (SE) | 62.71 (7.410) | 36.13 (12.622) |
| Median (Min, Max) | 50.92 (11.07, 185.93) | 38.27 (7.82, 79.06) |
|  |  |  |
| **EOT Week 53** |  |  |
| n | 36 | 5 |
| Mean (SE) | 27.06 (4.966) | 35.66 (7.890) |
| Median (Min, Max) | 20.43 (0.98, 130.24) | 36 (19.04, 63.04) |
| CFB: Mean (SE) | -35.64 (7.836) | -0.47 (4.754) |
| CFB: Median (Min, Max) | -25.24 (-178.36, 61.4) | -2.27 (-16.02, 11.22) |

CFB = Change from Baseline; SE=Standard Error; CI=Confidence Interval.

Missing data are assumed to be missing at random, and no imputation of missing values is performed.
