# Supplementary material for: 131I-LNTH-1095 Radioligand Therapy plus Enzalutamide versus Enzalutamide Alone in Men with PSMA-Avid Metastatic Castration-Resistant Prostate Cancer: A Phase II Study
Source: Clin Cancer Res. 2026 Mar 4;32(10):1973–82. doi: 10.1158/1078-0432.CCR-25-4948 (PMC13176818; doi:10.1158/1078-0432.CCR-25-4948)
Supplement: Supplementary Table S9 — Summary of 18F-piflufolastat SUVmax by Number of 131I-LNTH-1095 Therapy Cycles [file ccr-25-4948_supplementary_table_s9_suppts9.docx]

**Supplementary Table S9. Summary of ^18^F-piflufolastat Maximum Standardized Uptake Value (SUV_max_) by Number of ^131^I-LNTH-1095 Therapy Cycles**

| **Maximum SUV Overall** | **Number of ^131^I-LNTH-1095 Therapy Cycles** | | | |
| --- | --- | --- | --- | --- |
|  | **1** | **2** | **3** | **4** |
| **Baseline** |  |  |  |  |
| n | 1 | 11 | 11 | 19 |
| Mean (SE) | 79.06 | 62.35 (14.928) | 54.74 (11.430) | 61.93 (10.524) |
| Median (Min, Max) | 79.06 (79.06, 79.06) | 49.59 (11.07, 185.93) | 43.73 (13.67, 149.87) | 52.25 (7.82, 149.65) |
|  |  |  |  |  |
| **EOT Week 53** |  |  |  |  |
| n | 1 | 11 | 11 | 19 |
| Mean (SE) | 63.04 | 31.03 (11.374) | 22.96 (3.170) | 27.95 (6.788) |
| Median (Min, Max) | 63.04 (63.04, 63.04) | 17.29 (2.93, 130.24) | 24.27 (6.78, 43.85) | 21.6 (0.98, 125.44) |
| CFB: Mean (SE) | -16.02 | -31.33 (19.096) | -31.78 (11.774) | -33.97 (9.213) |
| CFB: Median (Min, Max) | -16.02 (-16.02, -16.02) | -7.54 (-178.36, 61.4) | -27.49 (-122.01, 7.57) | -15.32 (-127.51, 12.56) |

CFB = Change from Baseline; SE=Standard Error; CI=Confidence Interval.

Missing data are assumed to be missing at random, and no imputation of missing values is performed.
