# Supplementary material for: 131I-LNTH-1095 Radioligand Therapy plus Enzalutamide versus Enzalutamide Alone in Men with PSMA-Avid Metastatic Castration-Resistant Prostate Cancer: A Phase II Study
Source: Clin Cancer Res. 2026 Mar 4;32(10):1973–82. doi: 10.1158/1078-0432.CCR-25-4948 (PMC13176818; doi:10.1158/1078-0432.CCR-25-4948)
Supplement: Supplementary Table S10 — Treatment-Emergent Adverse Events [file ccr-25-4948_supplementary_table_s10_suppts10.docx]

| Randomized | ^131^I-LNTH-1095+ enzalutamide  N=76 | | Enzalutamide monotherapy  N=39 | |
| --- | --- | --- | --- | --- |
|  | All grades  n (%) | Grade≥3 ^a^ n (%) | All grades  n (%) | Grade≥3 ^a^ n (%) |
| Subjects with ≥1 TEAE | 73 (96.1) | 50 (65.8) | 37 (94.9) | 16 (41.0) |
| Fatigue | 57 (75.0) | 5 (6.6) | 21 (53.8) | 1 (2.6) |
| Dry mouth | 36 (47.4) | 0 | 1 (2.6) | 0 |
| Nausea | 45 (59.2) | 1 (1.3) | 13 (33.3) | 0 |
| Anemia | 32 (42.1) | 16 (21.1) | 6 (15.4) | 2 (5.1) |
| Back pain | 23 (30.3) | 4 (5.3) | 13 (33.3) | 3 (7.7) |
| Arthralgia | 24 (31.6) | 1 (1.3) | 18 (46.2) | 2 (5.1) |
| Decreased appetite | 37 (48.7) | 1 (1.3) | 7 (17.9) | 0 |
| Constipation | 13 (17.1) | 0 | 5 (12.8) | 0 |
| Diarrhea | 18 (23.7) | 0 | 10 (25.6) | 0 |
| Vomiting | 19 (25.0) | 0 | 6 (15.4) | 0 |
| Thrombocytopenia | 39 (51.3) | 29 (38.2) | 0 | 0 |
| Lymphopenia | 17 (22.4) | 13 (17.1) | 2 (5.1) | 1 (2.6) |
| Leukopenia | 15 (19.7) | 6 (7.9) | 1 (2.6) | 0 |

**Supplementary Table S10. Treatment-Emergent Adverse Events**

TEAE = treatment-emergent adverse event.

^a^The National Cancer Institute’s Common Terminology Criteria for Adverse Events (CTCAE) v5.0 was used for grading severity (0 = normal, 5 = death).
