# Supplementary material for: 131I-LNTH-1095 Radioligand Therapy plus Enzalutamide versus Enzalutamide Alone in Men with PSMA-Avid Metastatic Castration-Resistant Prostate Cancer: A Phase II Study
Source: Clin Cancer Res. 2026 Mar 4;32(10):1973–82. doi: 10.1158/1078-0432.CCR-25-4948 (PMC13176818; doi:10.1158/1078-0432.CCR-25-4948)
Supplement: Supplementary Table S11 — Most Common Serious Treatment-Emergent Adverse Events [file ccr-25-4948_supplementary_table_s11_suppts11.docx]

**Supplementary Table S11. Most Common Serious Treatment-Emergent Adverse Events**

| **^131^I-LNTH-1095+enzalutamide**  (N=76) | Enzalutamide monotherapy  (N=39) |
| --- | --- |
| Anaemia (2.6%) | Acute coronary syndrome (2.6%) |
| Pancytopenia (2.65) | Atrial tachycardia (2.6%) |
| Platelet count decreased/Thrombocytopenia(2.6%) | Gastrointestinal haemorrhage (2.6%) |
| Sepsis (2.6%) | Haematochezia (2.6%) |
| Fall (2.6%) | Abdominal infection (2.6%) |
| Dehydration (2.6%) | Hypokalaemia (2.6%) |
| Muscular weakness (2.6%) | Hypercalcaemia (2.6%) |
| Myelodysplastic syndrome (2.6%) | Arthralgia (2.6%) |
| Syncope (2.6%) | Back pain (2.6%) |
| Acute kidney injury (2.6%) | Osteonecrosis of jaw (2.6%) |
|  | Cerebrovascular accident (2.6%) |
|  | Spinal cord compression (2.6%) |
|  | Haematuria (2.6%) |
