# Supplementary figures and images for: 131I-LNTH-1095 Radioligand Therapy plus Enzalutamide versus Enzalutamide Alone in Men with PSMA-Avid Metastatic Castration-Resistant Prostate Cancer: A Phase II Study
Source: Clin Cancer Res. 2026 Mar 4;32(10):1973–82. doi: 10.1158/1078-0432.CCR-25-4948 (PMC13176818; doi:10.1158/1078-0432.CCR-25-4948)

**Supplementary Figure S1. Time to First New Anti-Cancer Treatment**


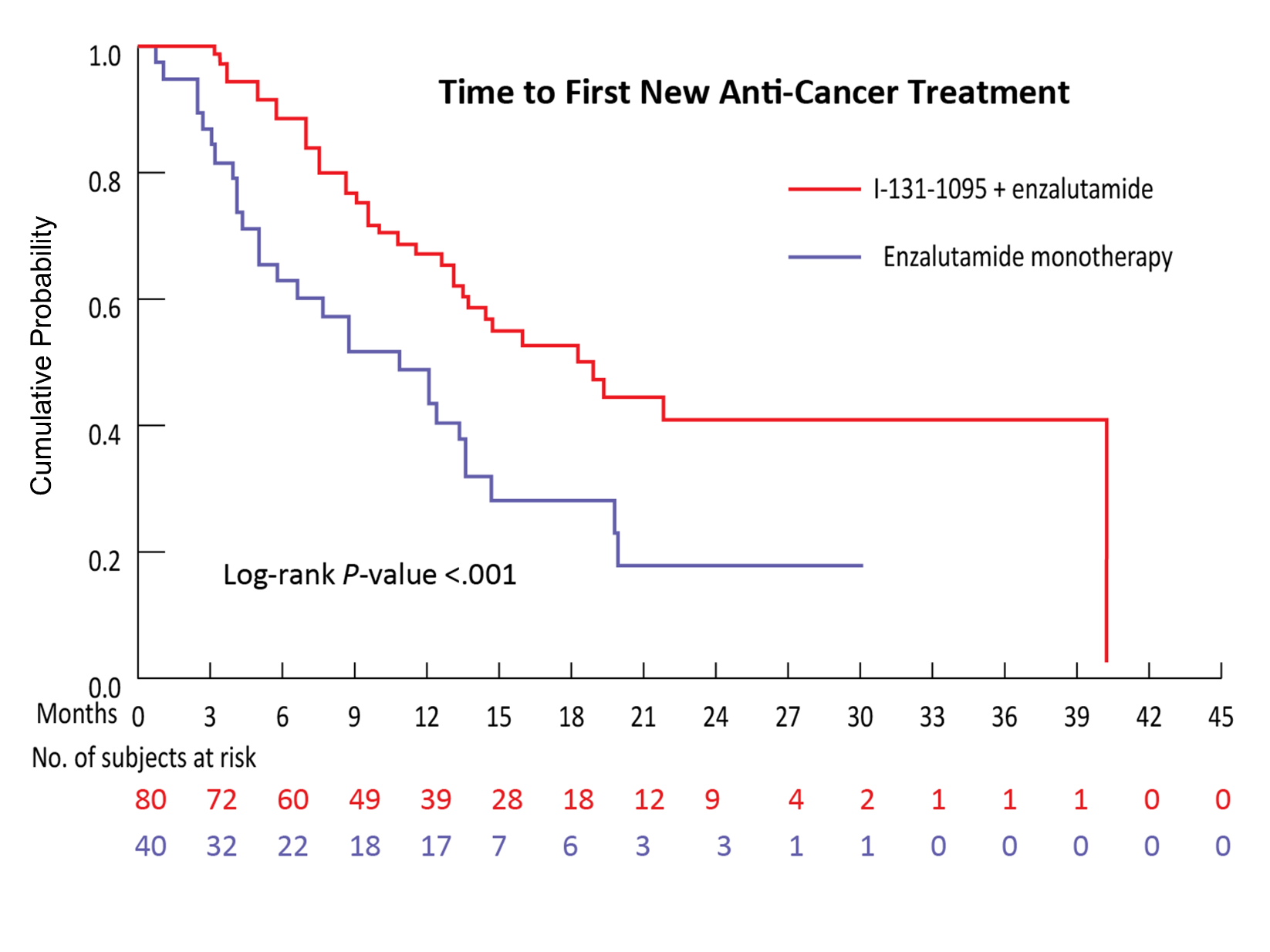

Supplement: Supplementary Figure S1 — Time to First New Anti-Cancer Treatment [file ccr-25-4948_supplementary_figure_s1_suppfs1.docx]
